# Immersive virtual reality for improving cognitive deficits in children with ADHD: a systematic review and meta-analysis

**DOI:** 10.1007/s10055-023-00768-1

**Published:** 2023-02-18

**Authors:** Niamh Corrigan, Costina-Ruxandra Păsărelu, Alexandra Voinescu

**Affiliations:** 1 Department of Psychology, University of Bath, Claverton Down, Bath, BA2 7AY UK; 2 Department of Clinical Psychology and Psychotherapy, The International Institute for the Advanced Studies of Psychotherapy and Applied Mental Health, Babe-Bolyai University, No.37, Republicii Street, 400015 Cluj-Napoca, Romania

**Keywords:** Virtual reality, Attention deficit-hyperactivity disorder (ADHD), Children, Cognition, Attention, Memory

## Abstract

Virtual reality (VR) shows great potential in treating and managing various mental health conditions. This includes using VR for training or rehabilitation purposes. For example, VR is being used to improve cognitive functioning (e.g. attention) among children with attention/deficit-hyperactivity disorder (ADHD). The aim of the current review and meta-analysis is to evaluate the effectiveness of immersive VR-based interventions for improving cognitive deficits in children with ADHD, to investigate potential moderators of the effect size and assess treatment adherence and safety. The meta-analysis included seven randomised controlled trials (RCTs) of children with ADHD comparing immersive VR-based interventions with controls (e.g. waiting list, medication, psychotherapy, cognitive training, neurofeedback and hemoencephalographic biofeedback) on measures of cognition. Results indicated large effect sizes in favour of VR-based interventions on outcomes of global cognitive functioning, attention, and memory. Neither intervention length nor participant age moderated the effect size of global cognitive functioning. Control group type (active vs passive control group), ADHD diagnostic status (formal vs. informal) and novelty of VR technology were not significant moderators of the effect size of global cognitive functioning. Treatment adherence was similar across groups and there were no adverse effects. Results should be cautiously interpreted given the poor quality of included studies and small sample.

## Introduction

Attention/deficit-hyperactivity disorder (ADHD) is a neurodevelopmental disorder, characterised by persistent inattention and/or hyperactivity/impulsivity, which interferes with normal functioning (American Psychiatric Association [APA] 2013). Although ADHD is found across the lifespan, ADHD is the most frequently diagnosed childhood developmental disorder (Polanczyk et al. 2014). Recent data from a meta-analysis indicated a pooled prevalence between 12.4 (Asian) and 15.9% (Black children and adolescents) (Cénat et al. 2022) and a worldwide ADHD prevalence of 5.9% among the youth population (Faraone et al. 2021). ADHD in children is also associated with a substantial national economic burden due to increased healthcare and educational costs (Schein et al. 2022), as well as academic underachievement (Holmberg and Bölte 2014), substance abuse disorder (SUD) (Ottosen et al. 2016), and social functioning impairments in later life (Sacchetti and Lefler 2017).

Treatment options for children with ADHD include pharmacological, nonpharmacological, and/or combined treatments (Dobrean et al. 2018). There are several different clinical guidelines (the National Institute for Health and Care Excellence, the European ADHD Guideline Group, the American Academy of Paediatrics) (the National Institute for Health and Care Excellence 2018; Wolraich et al. 2019; Coghill et al. 2021) for treating ADHD in children, which recommend different psychosocial (e.g. behavioural parent training) or combined treatments according to child’s age, ADHD severity or comorbidities presented. Pharmacological treatments, using stimulant (e.g. methylphenidate and dexamphetamine) or non-stimulant (e.g. atomoxetine, guanfacine) medication, are recommended for persistent and significant ADHD symptoms. Regarding psychosocial interventions, there are different options available for children and adolescents with ADHD which could be grouped according to the three waves of cognitive-behaviour therapies (Canu and Hilton 2022). First wave behaviour therapies are represented by behavioural parent training, behavioural classroom interventions, and by behavioural peer interventions. Second wave of behaviour therapies are represented by cognitive-behaviour therapy and training interventions (e.g. organisational skills training, social skills training, cognitive training, neurofeedback). Unlike the first wave therapies that focus on contingency management, second wave interventions aim to identify and use cognitive restructuring in order to reduce comorbid anxiety and depressive disorders (cognitive-behaviour therapy) and train different abilities that can be applied to other settings. Third wave behaviour therapies are mindfulness and dialectical behaviour therapy; they aim to improve emotion regulation and cognition through meditation and acceptance (Canu and Hilton 2022). From all these interventions, behavioural parent training, behavioural classroom interventions, behavioural peer interventions, and organisational skills training, are associated with moderate improvement in ADHD symptoms (Bikic et al. 2017; Evans et al. 2018; Groenman et al. 2022) while more research is needed in order to establish the efficacy of the other psychosocial interventions.

Despite the existence of evidence-based treatments for ADHD, a high number of children do not have access to such interventions. Significant barriers in treatment access exist and are related to costs, stigma, lack of knowledge, low treatment adherence (Wright et al. 2015; Kappi and Martel 2022). Given the existent barriers around current treatments, it is important to explore the potential of alternative interventions that can surpass gaps in treatment access. Last years of COVID-19 pandemic have worsened these challenges in treatment access for families with children diagnosed with ADHD (Golberstein et al. 2020; McGowan et al. 2020).

Given the extensive research on ADHD, many of the assumptions regarding current conceptualisation and treatment formulation based on the *Diagnostic and Statistical Manual of Mental Disorders* criteria (APA 2013) are being challenged (Sonuga-Barke et al. 2022). These new pathways in ADHD causes, heterogeneity in symptoms manifestation, shared genetics and neurobiology with other mental health problems, comorbid presentation with other conditions, could have important benefits for treatment decisions in ADHD (Sonuga-Barke et al. 2022).

Within the last decade, technological developments such as Internet-delivered interventions, assistive technology, mobile applications, and wearable devices have led to an increase in the implementation of digital technology to assess and treat a range of disorders (Păsărelu et al. 2017; Florean et al. 2020; Lehtimaki et al. 2021; Welch et al. 2022). Digital technologies, such as serious games, robots, or mobile applications have also been used as a tool to assess and treat ADHD symptoms in children (Choi et al. 2019; Păsărelu et al. 2020; Lakes et al. 2022; Kaimara et al. 2022). Among this new wave of digital technology is virtual reality (VR), defined as an advanced form of human–computer interaction created through the integration of computers (e.g. head-mounted displays [HMDs] such as Oculus or HTC Vive, more recently, body-tracking sensors, specialised interface devices, and 3D graphics) (Rizzo and Koenig 2017). The benefits of using VR applications for the education and support of children with developmental conditions such ADHD were highlighted in a recent systematic review (see Kaimara et al. 2022). VR can be subdivided into immersive and non-immersive experiences. The former refers to a computer-generated simulated world that occludes the user’s outward environment, whereas the latter refers to content delivered on a flat-screen monitor (e.g. desktop computer) with no occlusion of the user’s outward environment (Rizzo and Koenig 2017). Due to the occlusion of the external environment, immersive VR environments can be designed to closely replicate the cognitive demands of the real world, as such the tasks delivered in these environments are more ecologically valid than those delivered via non-immersive VR (Kober et al. 2012). Therefore, the expectations are that immersive VR would lead to greater therapeutical improvements (Voinescu et al. 2021b; Papaioannou et al. 2022). For this reason, immersive VR is considered a potential alternative intervention for children with ADHD. Already immersive VR classrooms have been created to assess (Rizzo et al. 2000; Iriarte et al. 2016; Neguț et al. 2017) and treat cognitive deficits in children with ADHD (Bioulac et al. 2020; David et al. 2021) with some promise. Recent meta-analyses have shown the effectiveness and validity of using VR in assessing attention deficits among children with ADHD (Parsons et al. 2019; Gilboa et al. 2021).

The evidence base for the effectiveness of VR-based interventions for the treatment of ADHD symptoms in children is steadily growing (Rizzo and Koenig 2017). In recent years, apart from employing VR in the assessment of ADHD with substantial advantages documented (e.g. more ecological, increased response accuracy; Neguț et al. 2017; Voinescu et al. 2021b; Papaioannou et al. 2022), VR-based interventions have been developed and tested in various forms (e.g. included in neurofeedback interventions, cognitive training, serious games) (Barba et al. 2019; Rodrigo-Yanguas et al. 2021). Preliminary evidence indicates that such interventions are associated with reduced inattentive symptoms, and mixed findings on impulsivity (Romero-Ayuso et al. 2021; Adabla et al. 2021). Recently several scoping and systematic reviews aimed to synthetise the evidence around the use of VR and/or serious games in children and youth with ADHD using other methodologies than RCTs (e.g. cross-sectional or case control designs). Results were suggesting initial supporting evidence in favour of using VR, but they included various VR platforms, some non-immersive (e.g. used screens and desktops) and serious video games (Adabla et al. 2021; Peñuelas-Calvo et al. 2022; Goharinejad et al. 2022; Rodrigo-Yanguas et al. 2022).

Due to increased heterogeneity among VR platforms, it is important to provide a synthesis of the literature to understand whether these new methods are effective in improving cognitive deficits in children with ADHD, and how they compare to currently recommended interventions. A recent meta-analysis of four studies concluded that immersive VR-based interventions were more effective in improving sustained attention and vigilance in comparison with controls receiving alternative treatment or no treatment (Romero-Ayuso et al. 2021). Although, immersive VR-based interventions were not more effective in improving impulsivity relative to controls. This meta-analysis provided important insights into how effective immersive VR-based interventions are for improving the primary cognitive deficits characteristic of ADHD. At present, no meta-analysis has investigated the effectiveness of immersive VR-based interventions for improving other specific domains of cognitive functioning that are associated with ADHD in children. This is an important line of enquiry given that ADHD in children affects a broad range of specific cognitive domains beyond attention and impulsivity such as memory, decision-making, and executive functioning (Coghill et al. 2014; Torgalsbøen et al. 2021). Furthermore, at present no review has investigated the effectiveness of immersive VR-based interventions in improving global cognitive functioning, by combining the outcome measures of the included studies. In children, global cognitive functioning is an indicator of academic performance (Tikhomirova et al. 2020), and social functioning (Tuerk et al. 2021), thus knowing whether immersive VR-based interventions are effective in improving global cognitive functioning may offer further insights into the potential positive implications VR-based interventions may have in other domains of life.

There is an additional dearth within the literature. It is not yet known what variables, if any, moderate the strength of effect size of cognitive outcomes. This information is important in order to guide future VR-based interventions and tailor such interventions to the unique profiles of users, however, so far, no data coming from a meta-analysis is available in order to guide the development of VR-based interventions for children with ADHD. Potential moderators include: the type of control group, intervention length, novelty of VR technology and variables relating to the demographics of the sample (e.g. age, gender, and diagnostic status of the sample). The type of control group can be a significant moderator as in the medical literature type of control group may produce different effects on the outcomes and is analysed accordingly (e.g. Bahar‐Fuchs et al. 2019; Voinescu et al. 2021a). For example, in the VR literature, Fodor et al. (2018) identified that type of control group was a significant moderator of effect size in a previous meta-analysis investigating the effectiveness of VR interventions on the severity of anxiety and depression (Fodor et al. 2018). No significant differences in effect size were observed between the VR group and active controls at post-intervention, whereas the effect size of the VR group was significantly larger compared with the passive controls. This would suggest that VR-based interventions are more effective than passive controls, who received no intervention, but equally as effective as currently used treatments received by active controls. This result highlights the importance of investigating the type of control group as a moderator to understand how VR interventions compare to groups receiving no treatment and groups receiving established treatments. This is relevant to clinical practice as any new interventions that are introduced to a patient group must be at least as effective as current treatments to ensure a cost-effective and efficient service.

Intervention length is another potential moderator that has been previously investigated concerning VR interventions with mixed results (Chen et al. 2014; Mekbib et al. 2020). This moderator has not been investigated for VR interventions implemented for children with ADHD, and it would be useful to know whether there is an optimum intervention length for the improvement of cognitive deficits. Novelty of VR technology was investigated in other reviews as it was considered this can affect the VR experience (Kourtesis et al. 2019). It was proposed that 2013 is the year for cut-off between old generation HMDs and new because in 2013 the first new generation HMD prototype Oculus Development Kit 1 was released (Kourtesis et al. 2019). Research links novelty of HMDs with increased simulator sickness and reduced study drop-outs (see Kourtesis et al. 2019 for a full review). To address this, in the current meta-analysis we planned to investigate if old versus new VR technology moderated the improvements of cognitive performance. Because research links novelty of HMDs and treatment adherence, we also addressed the moderating effects of these factors, by comparing differences between drop-outs as a measure of adherence in the VR groups versus control groups. We also reported any adverse effects described by authors. Simulator sickness is one adverse effect that is documented in the VR literature and is described to occur during exposure in VR (e.g. general discomfort, fatigue, headache, eye strain, stomach awareness, nausea, dizziness, vertigo, and burping, sweating, blurred vision Kennedy et al. 1993; Kolasinski 1995; Kim et al. 2018).

Moderator variables relating to the demographics of the sample are also relevant. For example, participant age has been highlighted as a significant moderator in a meta-analysis investigating the effectiveness of VR-based interventions for children with cerebral palsy, with effect sizes on arm function and ambulation for younger children significantly larger than older children (Chen et al. 2018). The authors suggested that younger children may be more adaptable than older children, and so can make larger improvements than older children regarding cognitive functioning. It would be interesting to see whether this finding applies to children with ADHD. We also accounted for the type of diagnosis. In short, psychiatric diagnosis is made by clinical professionals following established guidelines (e.g. according to the International Classification of Diseases 10th Revision, ICD-10; WHO 2001, and/or the Diagnostic Statistical Manual of Mental Disorders, DSM-5; APA 2013). However, in published studies it is not unusual to include participants with elevated symptoms that meet the cut-off criteria for ADHD as measured on several scales, but without adhering to the rigorous standards of DSM-5 or ICD-10. This situation is acknowledged in related fields (e.g. people with mild cognitive impairment, dementia) where similar subgroup analyses were conducted to account for formal and informal and scale-based diagnosis (e.g. Papaioannou et al. 2022). Furthermore, according to research there may be a long time until children with significant ADHD symptoms receive an ADHD diagnosis. Specifically, data coming from a large study conducted with caregivers of children with ADHD indicated that the average duration between the first doctor visit to a formal diagnosis is 10.8 months in EU countries and can be up to 18.3 months in the UK for example (Fridman et al. 2017). As so, even if the child could then meet the criteria, they await formal classification.

This review aims to address gaps in the literature by assessing the effectiveness of immersive VR-based interventions on specific cognitive domains beyond those typically associated with ADHD in children, as well as global cognitive functioning. Additionally, this review aims to conduct moderation analyses with relevant variables, such as: type of control group, intervention length, novelty of VR technology, participant age and diagnosis status and address important questions concerning immersive VR interventions adherence. Namely, we aimed to answer following research questions: (1) Are immersive VR-based interventions effective in improving cognitive deficits in children with ADHD? (2) What are the factors that influence the effect sizes?; and (3) Are VR interventions feasible in terms of treatment adherence and safe?

## Methodology

### Study design

A meta-analysis and systematic review were conducted according to the Cochrane Handbook for Systematic Reviews of Interventions (Higgins et al. 2019), and the PRISMA Declaration guidelines (Page et al. 2021) to address our research questions. This systematic review was registered in the International Prospective Register of Systematic Reviews (PROSPERO CRD42021258310).

### Search strategy

A literature search was conducted to identify relevant records. A search strategy was devised using the PICO framework and Boolean Logic. The search string included terms related to ADHD (ADHD OR “attention deficit” OR “hyperactivity disorder”) combined with terms related to the population investigated (children), intervention (“virtual reality” OR VR OR “virtual environment” OR immersive) and outcomes (“cognition” OR “cognitive” OR “attention” OR “sustained attention” OR “impulsivity” OR “cognitive impulsivity” OR “executive function” OR “vigilance” OR “distractibility” OR “inhibition” OR “dual task” OR “inhibitory control”). Searches were completed in PsycINFO, Web of Science with MEDLINE, Embase, and Cochrane Library’s Central Register of Controlled Trials (CENTRAL) databases through April 2021 and updated in October 2022. These are major healthcare data bases with an excellent cover of VR and ADHD interventions literature which were used in similar studies (e.g. Bahar‐Fuchs et al. 2019; Voinescu et al. 2021a). At full-text screening, the list of references of the records was screened independently by two researchers (NC and AV) to detect any other relevant studies that did not appear in the initial database search.

### Eligibility criteria

The criteria for the inclusion of studies in the meta-analysis is outlined in Table 1 using the PICO framework. Randomised controlled trials (RCTs) that compared an immersive VR-based intervention with a control group were included. Clinical trial protocols and conference papers that did not present results were excluded. We had no publication date restrictions and included studies published in any years if they meet our eligibility criteria. Studies included were available in full-text and published in English. We included children and youth population. Full details concerning our eligibility criteria can be found in Table 1.

**Table 1:** Criteria for the inclusion of studies in the meta-analysis

PICO Field	Criteria
Population	Included participants were children and adolescents aged ≤ 18. 18-year-olds were included on the basis that previous meta-analyses (Romero-Ayuso et al. 2021), scoping reviews (Adabla et al. 2021), and global prevalence reviews (Faraone et al. 2021) investigating children with ADHD have included 18-year-olds. Studies where participants had a formal diagnosis of ADHD according to the International Classification of Diseases 10th Revision (ICD-10) (WHO 2001), the DSM-5 (APA 2013), or any of their previous iterations were included. Participants who did not have a formal ADHD diagnosis, but displayed ADHD-like symptoms (i.e. inattention, hyperactivity) as observed by an external party (e.g., clinician, research personnel), or as assessed by a validated measure were also included. Excluding participants based on an absence of formal diagnosis was deemed inappropriate given that access to ADHD diagnostic services is reported to be difficult for caregivers of children with attention and/or hyperactivity problems (Fridman et al. 2017). As such an absence of a formal diagnosis does not mean the participants do not have ADHD, rather they may lack access to diagnostic services
Intervention	Any immersive VR-based intervention was included where the participant’s outward environment is occluded using a head-mounted display (HMD) or the integration of two or more computers (body-tracking sensors or specialised interface devices with 3D graphics). Non-immersive interventions where the content was delivered on a flat-screen monitor with no occlusion of the user’s outward environment were excluded
Comparator	Studies using no treatment/waiting list, where participants received no intervention were included under the term of passive control groups. Wait-list control groups were included under the umbrella term of passive control groups as participants are withheld treatment and are offered treatment at the end of the study (e.g. Bahar‐Fuchs et al. 2019). Studies using an active comparator group, where participants received an intervention with similar levels of contact with research personnel and a similar number of sessions as the intervention group (e.g. psychotherapy or non-immersive VR) were included. Also, as per clinical guidelines, medication was also considered as an active comparator group
Outcome	Included studies used standardised outcome measures assessing either global cognitive functioning or any specific domain of cognitive functioning. Examples of eligible outcome measures include any Continuous Performance Test (CPT) (e.g. Tests of Variable Attention [TOVA], or the Integrated Visual and Auditory CPT [IVA]), or any subset of the Wechsler Intelligence Scale for Children-IV [WISC-IV] (e.g. Working Memory Index [WMI])

### Data extraction

Two independent researchers extracted data. The search results from each database were exported to EndNote Compressed Library (version X9.2, Clarivate Analytics 2019). Afterwards, the abstracts were screened against the eligibility criteria, followed by a full-text screening. At full-text screening when information relating to the inclusion criteria was not clearly reported in the paper the authors were contacted via email for clarification. Once a final list of included records was identified the following variables were extracted: study identification data (i.e. authors and year of publication), intervention aims, outcome measures, total sample size, number of participants per condition, the diagnostic status of the sample, novelty of VR technology, participants mean age, percentage of male participants, medication usage within the sample, type of intervention condition, type of control condition, length of intervention and control group, information concerning adverse effects, e.g. simulator sickness, number of participant drop-outs at the end of intervention and post-intervention results.

### Effect size calculation and heterogeneity

The statistical analyses were conducted using Comprehensive Meta-Analysis (version 3, Borenstein et al. 2013). To answer the first research question, between-group effect sizes were calculated using Hedges’s *g* with the following cut-off points: small effect (*g* = 0.20 to 0.50), moderate effect (*g* = 0.50 to 0.80), large effect (*g* > 0.80) (Cohen 1988). To compute effect sizes the sample size, alongside the mean scores and standard deviation at post-intervention were used. Where the mean and standard deviation scores were not reported, Hedges’s *g* values were calculated using exact* t*, *F*, and* p* values. Effect sizes were computed for each study using a random-effects model, and the study was used as the unit of analysis, whereby positive effect sizes indicated the advantage of the intervention group and negative effect sizes indicated the advantage of controls. For studies with multiple conditions, all relevant experimental/control groups were combined into a single experimental/control group. To assess the effects of immersive VR on individual cognitive domains a between-group analysis was conducted to assess attention and memory by combining the relevant outcome measures for these domains. A between-group analysis was also conducted to assess global cognitive functioning by combining all outcome measures included in the study that assessed any area of cognitive functioning.

To assess for heterogeneity of the effect sizes the homogeneity *Q* test and the *I*^2^ index was used. The homogeneity *Q* test was used to assess the statistical significance of the heterogeneity, where significant heterogeneity is *p* < 0.10 (Deeks et al. 2019). The level of heterogeneity was estimated using the *I*^2^ index with the following cut-off points: low (*I*^2^ < 40%), moderate (*I*^2^ = 40% to 60%), substantial (*I*^2^ = 60% to 90%), considerable (*I*^2^ > 90%) (Deeks et al. 2019).

For our second research question, moderation analyses were conducted using a mixed-effects model for categorical data and meta-regressions to investigate the potential source of heterogeneity from the continuous variable’s intervention length and participant age. A subgroup analysis was conducted to investigate the potential source of heterogeneity from the following categorical variables:

- Type of control intervention for the comparisons of the intervention group: passive versus active.
- Novelty of VR technology: older versus newest HMDs
- Diagnostic status of the sample: formal ADHD diagnosis versus ADHD-like symptoms without a formal diagnosis.

To address our third research question concerning the adherence to VR interventions versus controls we expressed results as a risk ratio (RR) with a 95% CI.

### Study quality (Risk of bias assessment)

A quality appraisal of each study was conducted independently by two study authors (NC and AV) using the Cochrane 'Risk of bias 2’ (RoB 2) tool (Sterne et al. 2019) and disagreements were resolved with CP. Based on the criteria from the Rob 2 tool, studies were categorised as being at low risk of bias (Green (+), high risk of bias (Red (−) and having some concerns (Yellow, (?). The assessments were conducted for five individual domains: bias arising from the randomisation process, bias due to deviations from intended interventions, bias due to missing outcome data, bias in measurement of the outcome, bias in selection of the reported result and an overall bias.

### Publication bias

The Duval and Tweedie (2000) trim-and-fill procedure was used to investigate publication bias. This method removed studies with extreme effect sizes that caused funnel plot asymmetry and used the ‘trimmed’ funnel plot to estimate the true centre of the funnel to give an unbiased estimate of the effect size.

## Results

### Study selection

**Fig. 1:**
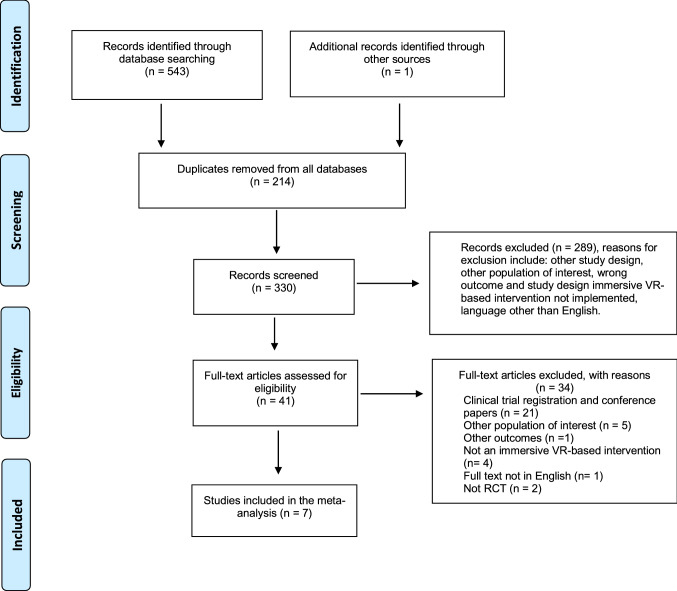
PRISMA flow diagram

We identified 543 records through database searching whilst one additional record (Bul et al. 2016) was identified through searching the list of references of the records obtained at the full-text screening. After excluding 214 duplicates, we screened a total of 330 records based on their title and abstract and excluded 289 records. We assessed the remaining 41 records in full and excluded 34 records. We included in the meta-analysis seven studies (see Fig. 1).

### Description of studies

Table 2 provides descriptive information about the included studies. All studies were randomised controlled trials (RCTs), three of which explicitly mention an element of randomisation (Cho et al. 2004; Bioulac et al. 2020; Tabrizi et al. 2020; Skalski et al. 2021), the other half (Lee et al. 2001; Cho et al. 2002; Kim et al. 2020) did not explicitly mention randomisation in-text but were confirmed to be randomised studies by the original authors via email correspondence. All studies, apart from Tabrizi et al. (2020), explicitly mention the use of a head-mounted display (HMD). Tabrizi et al. (2020) confirmed they used an HMD via email correspondence. In addition to the use of an HMD, Cho et al. (2002) and Cho et al. (2004) integrated neurofeedback training into the intervention. Skalski et al. (2021) used two intervention groups, VR with and without distractors. Three studies used recent VR technology such as HMDs like HTC Vive (Kim et al. 2020; Tabrizi et al. 2020; Skalski et al. 2021) while others used an older version of HMDs (Lee et al. 2001; Cho et al. 2002, 2004; Bioulac et al. 2020).

**Table 2:** Description of the included studies

Author	Participants	Measures	Intervention Group	Comparison Group	Outcome
Bioulac et al. (2020)	*N* = 48 10 girls 38 boys, 7–11 years (*M* = 8.9, *SD* = 1.2 years), diagnosed with ADHD according to the DSM-IV Exclusion criteria: children with comorbid conditions and cognitive functioning < 85. No information concerning accompanying/previous treatment	ADHD-RS Symptom Inventory; Continuous Performance Test (CPT); Virtual Classroom Assessment (measuring the number of correct hits, and the number of commission errors)	Participants wore an HMD which displayed a virtual classroom. Participants completed a letter identification task whilst experiencing visual and audio distractors *n* = 16. No. of sessions = 12. Duration of each session = 30 min. Frequency of sessions = twice a week. Intervention period = 6 weeks. Drop-outs: 3	*Psychotherapy Intervention:* Participants underwent individual psychotherapy, focusing on primary ADHD, emotional symptoms of ADHD, and the impact of ADHD on daily life (notably on self-esteem) *n* = 16. No. of sessions = 12. Duration of each session = 30 min. Frequency of sessions = twice a week. Intervention period = 6 weeks. Drop-outs: 2	*ADHD-RS Symptom Inventory* There were significant differences between the groups on ADHD-RS total, ADHD-RS inattention, and ADHD-RS hyperactivity, from baseline to post-treatment. The psychostimulant treatment group exhibited significantly lower ADHD-RS total, ADHD-RS inattention, and ADHD-RS hyperactivity than the other two groups *CPT* There were significant differences for all groups on the number of omission errors and commission errors from baseline to post-treatment. Post-treatment there was no significant differences in the number of omission errors between the VR-based group and the psychostimulant group. Post-treatment the number of commission errors in the psychostimulant treatment group was significantly higher than in the VR-based group
Bioulac et al. (2020) cont				*Psychostimulant Treatment:* Participants were treated with long-acting methylphenidate (QUASYM®) and completed a clinical interview once every 2 weeks for 8 weeks with a posologist. The posology of the long-acting methylphenidate was adapted according to the clinical response *n* = 19. Drop-outs: 4	*Virtual Classroom Assessment* The number of correct hits post-treatment was significantly higher for the VR-based intervention than the psychotherapy treatment group, but not significantly higher than the psychostimulant treatment group. Post-treatment, the number of commissions in the VR-based intervention group was significantly lower than the psychostimulant group, but not significantly lower than the psychotherapy group
Cho et al. (2002)	*N* = 50, ages 14–18 years, with a history of learning difficulties, inattentiveness, impulsivity, and hyperactivity. Other demographic information not reported. No information concerning accompanying/previous treatment	Continuous Performance Test (CPT)	*Experimental Group 1: Cognitive Training* Participants underwent two cognitive training courses utilising an HMD: Virtual Reality Comparison Training (VRCT) and Virtual Reality Sustained Attention Training (VRST). During the VRCT course, participants viewed two 3D objects and decided whether the objects were identical or not. During the VRST course, participants viewed an Arabic numeral and were encouraged to respond by pressing a mouse button when the numeral ‘0’ was presented after any digit other than ‘8’	*Group 1: Cognitive Training* Participants performed the same task as Experimental Group 1 using only a computer monitor *Group 2: Neurofeedback Training* Participants performed the same task as Experimental Group 2 using only a computer monitor	Both the experimental and control groups showed a significant increase in the number of correct responses on the CPT post-treatment. Post-treatment the neurofeedback experimental and control groups scored high in the number of correct responses on the CPT than the cognitive training experimental and control groups, although this difference was not significant. The response sensitivity of both the experimental groups improved slightly more than the control groups, although this difference was not significant
Cho et al. (2002) cont			*Experimental Group 2: Neurofeedback Training* Participants underwent the same training outlines in Lee et al. (2001) *n* for experimental group 1 = 10. *n* for experimental group 2 = 10. No. of sessions = 8. Duration of each session = 20 min. Drop-outs: 0	*Control Group 3:* Participants underwent no training during the same period. No other information was reported *n* for Control group 1 = 10. *n* for Control group 2 = 10. *n* for control group 3 = 10. No. of sessions = 8. Duration of each session = 20 min. Drop-outs: 0	
Cho et al. (2004)	*N* = 28, all were boys, ages 14–18, with no official ADHD diagnosis Subjects had a history of learning difficulties, inattentiveness, impulsivity, and hyperactivity. No information concerning accompanying/previous treatment	Continuous Performance Test (CPT)	Participants underwent the same intervention outlined in Lee et al. (2001) and Cho et al. (2002) (experimental group 2: neurofeedback training) *n* = 10. No. of sessions = 8. Duration of each session = 20 min. Intervention period = 2 weeks. Drop-outs: 0	*Non-immersive-VR Group:* Completed the same task as the VR-based intervention group, with only a computer monitor *n* = 9. Drop-outs: 0 *Control Group:* Participants received no intervention *n* = 9. Drop-outs: 0	At post-intervention, the VR-based intervention group had a significantly higher number of hits on the CPT than both control groups. At post-intervention, the VR-based intervention group was marginally significantly faster to react in the CPT than both control groups. All groups showed a decrease in perceptual sensitivity and a reduction in commission errors at post-intervention, and although the VR group demonstrated the lowest scores for both measures this difference was not significant. All groups demonstrated a reduction of omission errors at post-intervention; however, the omission errors of the VR group were significantly lower than both control groups
Kim et al. (2020)	*N* = 40, 5 girls and 35 boys, ages 8–10 years, with ADHD diagnosed by a psychiatrist. Included only children that were not using medication or other treatments during the intervention/game	Advanced test of attention (ATA); Interactive metronome (IM)	Participants wore a HoloLens (this is a self-contained mobile HMD), and then participate in a series of eye-contact training games. The first five sessions, the second five sessions, and the third five sessions had different levels of difficulty ranging from easy to difficult *n* = 20. No. of sessions = 15. Duration of sessions = 30 min. Intervention period = 6 weeks. Drop-outs: 0	No intervention was received. *n* = 20. Drop-outs: 0	*ATA* The omission and commission errors of the VR-based intervention group decreased significantly from pre- to post-intervention, but those of the control group exhibited a small decrease that was not significant. The omission errors of the VR-based intervention group at post-test were significantly lower than the control group, however, the commission errors of the VR-based intervention group were not significantly lower than the control group *IM* The mean response times decreased significantly for both groups from pre- to post-intervention. At post-intervention, the VR-based intervention group were significantly faster to respond than the control group in the hand, feet, both hands, both feet, right side, and bilateral cases
Lee et al. (2001)	*N* = 20, all participants were boys with impulsivity and attention problems. Other demographic information not reported. No information concerning accompanying/previous treatment	Continuous Performance Test (CPT)	Participants wore a head-mounted display (HMD) displaying a dinosaur egg on a desk. Electrodes behind the participant’s ears collected biofeedback data. The dinosaur egg fractured, and participants were instructed to reassemble the egg. The participants were also presented with information about the dinosaur and were asked multiple-choice questions about the dinosaur *n* = 10. No. of sessions = 10. Duration of each session = 10 min. Intervention period = 2 weeks. Drop-outs: 0	No intervention was received. No other information is reported. *n* = 10. Drop-outs: 0	The VR-based intervention group showed improvements in perceptual sensitivity from pre- to post-intervention compared to the control group. The authors do not indicate whether this improvement was statistically significant. Both the experimental and control groups showed reductions in omission and commission errors from pre- to post-intervention. The VR-based intervention group showed a larger decrease in commission and omission errors post-test in comparison with the control group, however, the authors do not indicate whether this difference was statistically significant
Skalski et al. (2021)	*N* = 87, 12 girls and74 boys, 9–15 years (*M* = 12.75, *SD* = 1.7 years), diagnosed with ADHD according to the DSM-V Exclusion criteria: neurological condition, intellectual disability, fast wave amplitude within the norm (< 30 μV in the 18–30 Hz band) in the quantitative EEG (QEEG)	The Short Form of the Mackworth Clock Task (SFMCT); The Visual Search Task (VST); The Multitasking Test (MT)	Participants were randomly distributed into two intervention groups: 3D VR hemoencephalographic biofeedback (HEG BFB) with and without distractors. In VR participants were transferred into a virtual room fitted with a Computer, furniture, paintings, plants and windows and observed the flash game image on a computer screen located in the virtual room. In the distractor condition participants were immersed directly into the environment, while in the no distractor condition the participants were immersed into the environment with limited objects available. HMD was a HTC VIVE *n* = 57. No. of sessions = 10. Length of sessions = 45 min. Intervention period = 10 weeks. Drop-outs: 3	Participants from the traditional 2D HEG BFB training Sessions (HEG BFB standard) group observed flash games on a 21- Inch TV screen. The room was additionally fitted with furniture, paintings, plants and windows. *n* = 30. No. of sessions = 10. Length of sessions = 45 min. Intervention period = 10 weeks. Drop-outs: 0	Significant improvements in favour of the VR with and distractor groups versus traditional 2D HEG BFB on all outcomes: omission and commission errors (measured with SFMCT), RT slope in visual search (VST), RT in single task and multi-task (MT)
Tabrizi et al. (2020)	*N* = 48, 16 girls and 32 boys, ages 7–12 years, with ADHD diagnosed by a psychiatrist. Participants had an IQ > 81. All participants were instructed to stop medication for 3 weeks before the study commenced. Included children that stopped medication at least 3 weeks before intervention	The digit span subscale, which is a subset of the verbal scale of Wechsler Children's Intelligence Test	Participants wore an HMD and completed a series of tasks within a virtual classroom with increasing difficulty (the number of targets and distractors increased as the session progressed) *n* = 16. No. of sessions = 10. Length of sessions = 3 min. Drop-outs: 0	*Medication Group* Participants received treatment with medicines, such as Ritalin, atomoxetine, and dexamphetamine. No. of participants = 16 *Control group* No intervention was received; participants did not take medication. *n* = 16. Drop-outs: 0	Participants in the VR-based intervention and medication group made significant improvements in memory from baseline to post-intervention. At post-intervention, the VR-based intervention scored significantly higher on the digit span subscale than both control groups. At post-intervention, the medication group scored significantly higher on the digit span subscale than the control group. At follow-up, the VR-based intervention scored significantly higher on the digit span subscale than both control groups. At follow-up, the medication group scored significantly higher on the digit span subscale than the control group

*N* the total number of participants, *n* number of participants in a fraction of the total sample, *M* average mean, *SD* standard deviation

The total number of participants included in the meta-analysis was 321, 149 of which were assigned to an experimental group whilst the remaining 172 were assigned to a comparator group. Only two studies reported data on the existence of prior/current treatment. For example, Kim et al. (2020) included only children that were not using medication or other treatments during the intervention and Tabrizi et al. (2020) included children that stopped medication at least 3 weeks before intervention. Cho et al. (2002) and Cho et al. (2004) used a passive control group, where participants received no intervention and an active control group where participants completed the same tasks as the intervention group using non-immersive technology; however, in case of Cho et al. (2002) sufficient data to compute effect sizes were only for the active controls. Tabrizi et al. (2020) used one active control group with pharmacotherapy and one passive control group that did not receive any medication. Kim et al. (2020) and Lee et al. (2001), used only passive control groups, where participants received no intervention. Bioulac et al. (2020) used two active control groups: psychotherapy and pharmacotherapy. Here, children in the pharmacotherapy group received two clinical interviews a week for the duration of the study with a posologist, who adapted the posology of the medication per the participant’s clinical response. Usual pharmacotherapy for children with ADHD would not include regular clinical interviews with a specialised posologist (NICE 2018). Skalski et al. (2021) used an active control group that received hemoencephalographic biofeedback. Of the studies that reported the gender of the sample, 71% (*n* = 227) were male. Bioulac et al. (2020), Kim et al. (2020), Skalski et al. (2021) and Tabrizi et al. (2020) recruited participants with a formal ADHD diagnosis, whilst the remaining studies recruited participants with persistent inattention and hyperactivity problems who did not have a formal ADHD diagnosis. Tabrizi et al. (2020) was the only study to include a follow-up assessment 2-months after post-intervention.

**Fig. 2:**
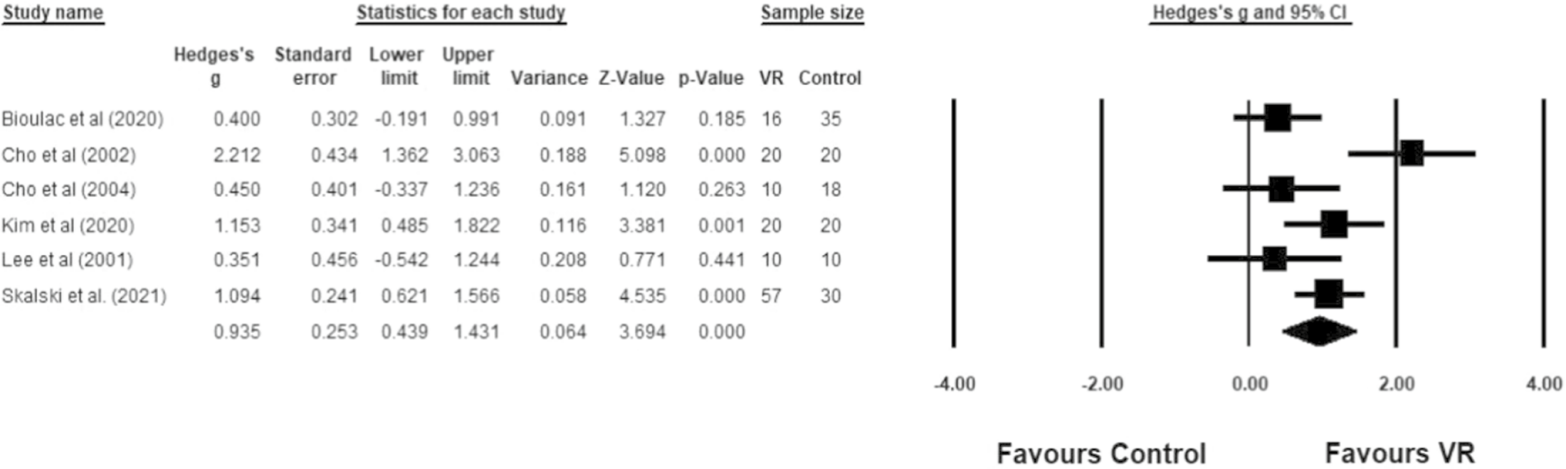
Comparison between VR group and control group on attention measures

For the first research question, a between-group analysis was conducted by comparing the VR groups and control groups on attention. A significantly large effect size was found in favour of the VR group (*g* = 0.94, 95% CI [0.44, 1.43], *z* = 3.69; *p* < 0.001). There was significant substantial heterogeneity in the results (*Q* (5) = 15.75, *p* < 0.01; *I*^2^ = 68.26%). Figure 2 shows the forest plot alongside the statistics for each study.

As only one study (Tabrizi et al. 2020) included memory outcomes a meta-analysis could not be performed. Instead, the means, *SDs* and computed Hedge’s *g* value are reported. The VR group (*M* = 11.19, *SD* = 1.72) outperformed the control group (*M* = 7.00, *SD* = 2.51) with a significantly large effect size (*g* = 1.81; *p* < 0.001).

**Fig. 3:**
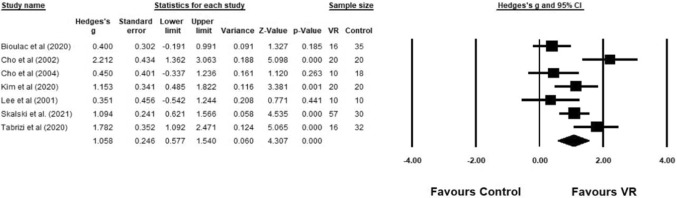
Comparison between VR group and control group on all outcome measures

A between-group analysis with all seven studies was conducted by comparing the VR groups and control groups on all outcomes as an indication of global cognitive functioning. A significantly large effect size was found in favour of the VR group (*g* = 1.06, 95% CI [0.58, 1.54], *z* = 4.31; *p* < 0.001). There was evidence of significant substantial heterogeneity in the results (*Q* (6) = 20.85, *p* = 0.002; *I*^2^ = 71.23%). This was investigated further using moderation analyses. Figure 3 shows the forest plot alongside the statistics for each study.

As only two studies reported data on the existence of prior/current treatment we performed an additional sensitivity analysis to see whether the effect would change in studies where children were withheld treatment. Results showed a large effect size in favour of the immersive VR group (*g* = 1.46, 95% CI [0.85, 2.07], *z* = 4.65; *p* < 0.001) with low heterogeneity in the results (*Q* (1) = 1.65, *p* = 0.20; *I*^2^ = 39.26%).

### Moderation analysis

To investigate our second research question several subgroup analyses were conducted. The results from the between-group analysis for global cognitive performance revealed significant substantial heterogeneity. This was explored further by performing a subgroup analysis for categorical variables and meta-regressions for numerical variables outlined in the method section.

Results from a meta-regression (see Table 3) revealed that intervention length did not significantly moderate the effect size of global cognitive functioning (*β* = − 0.001, 95% CI [− 0.004, 0.002], *z* = − 0.54; *p* = 0.59). A second meta-regression was conducted with participant’s mean age, where participants mean age was not reported the median age of participants was used. Only six studies were included in this meta-regression as Lee et al. (2001) did not report any information on participant age. Results revealed that participant age did not significantly moderate the effect size of global cognitive functioning (*β* = 0.04, 95% CI [− 0.15, 0.22], *z* = 0.39; *p* = 0.70).

**Table 3:** Meta-regression of global cognitive functioning with numeric variables for intervention length and participant age

Moderator	*k*	*β*	*SE*	95% CI	*Z*	*p*
Intervention length	7	− 0.001	0.002	[− 0.004, 0.002]	− 0.54	0.59
Participant age	6	− 0.04	0.09	[− 0.15, 0.22]	0.39	0.70

*k* number of studies included in the analysis, *β* meta-regression coefficient, *SE* standard error, *95% CI* 95% confidence interval around the weighted mean effect size, *Z* value for testing statistical significance for one coefficient

The subgroup analysis revealed that type of control (passive vs. active) did not moderate the effect size of global cognitive functioning (see Table 4). There were no significant differences in the effect size associated with global cognitive functioning between the intervention group and active controls. We also investigated the moderation effects of novelty of immersive VR technology (newest and older date of HMD). Results showed that novelty of VR technology was not a significant moderator of the treatment effects (see Table 4). Similarly, the diagnostic status of the sample (formal ADHD diagnosis vs. participants with ADHD-like symptoms but no formal diagnosis) was not a significant moderator of global cognitive functioning (see Table 4).

**Table 4:** Subgroup analyses of global cognitive functioning with categorical variables for type of control group, diagnostic status of the sample and novelty of VR technology (mixed-effects model)

Moderator	*k*	*g*	*p*	*I*^*2*^	*95%* CI	*Q*_*b*_	*p*
Active controls/passive controls	5	0.95	0.003	75.91	[0.33, 1.58]	0.28	0.60
	4	1.30	0.03	85.25	[0.17, 2.44]		
Formal ADHD diagnosis/ADHD-like symptoms without a formal ADHD diagnosis	4	1.09	< 0.001	67.00	[0.57, 1.61]	0.02	0.90
	3	1.00	0.10	82.92	[− 0.17, 2.18]		
Newest VR technology/older VR technology	3	0.89	< 0.001	49.27	[0.42, 1.35]	0.39	0.53
	4	1.21	0.008	80.09	[0.31, 2.11]		

*k* number of studies, *g* Hedge’s *g*, *I*^*2*^ Heterogeneity within study, *Q*_*b*_ Heterogeneity between studies

### Adherence to treatment and safety of immersive VR-based interventions

For our third research question, five studies reported no drop-outs at the end of treatment (Cho et al. 2002, 2004; Kim et al. 2020; Lee et al. 2001; Tabrizi et al. 2020). Skalski et al. (2021) had 3/60 drop-outs in the immersive VR group and 0/30 in the control group. Bioulac et al. (2020) reported 3/19 drop-outs in the VR group and 6/41 in the control group. Results showed no significant differences between the immersive VR and control groups in the number of participant drop-outs at the end of intervention, *RR* = 1.45, 95% CI [0.45, 4.61], *z* = 0.62, *p* = 0.53). Only two studies documented the occurrence of adverse effects. Bioulac et al. (2020) reported that no adverse effects occurred. Similarly, Kim et al. (2020) reported that in the VR group overall all participants responded negative to simulator sickness questions, with the exception of one child that responded positive to the question “My head became heavy. (Fullness of head)”. None of the other studies reported detailed data for both VR and control groups, so a meta-analysis was not conducted.

### Risk of bias assessment

**Fig. 4:**
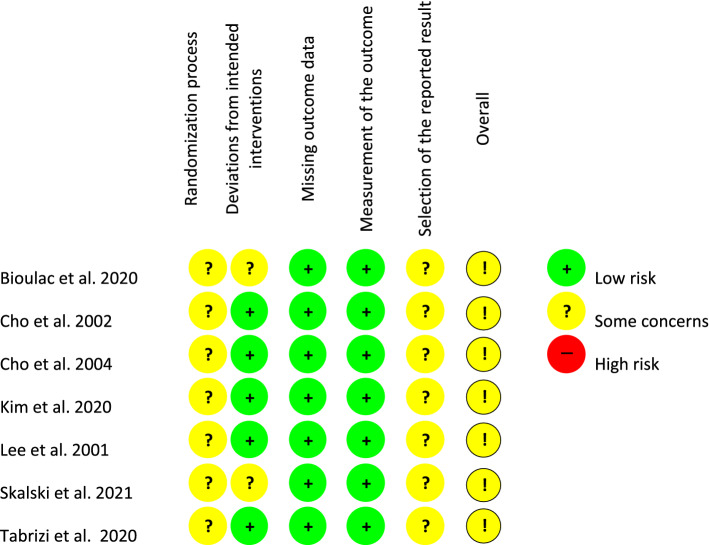
Risk of bias judgements for the included studies

All studies were judged to be of unclear risk of bias concerning the randomisation process, as all studies failed to report sufficient information about how randomisation was conducted and whether the allocation sequence was concealed before the study commenced. For example, most studies included a general statement such as “the children were randomly assigned”. This was considered insufficient to judge if the participants were allocated to groups using a random component. All the studies except Bioulac et al. (2020) and Skalski et al. (2021) were judged to be at low risk of bias due to deviations from intended intervention (effect of adhering to intervention). This was because the domain’s questions were not applicable to any of the studies as no statements were made that the assessment will address the imbalance of non-protocol interventions between the intervention groups and for most studies no drop-outs occurred. All the studies were judged to be at a low risk of bias due to missing data as there were no drop-outs from pre-test to post-test and outcome data was available for all or nearly all participants at the end of the intervention. All studies were judged to be at low risk of bias due to outcome measurement, and all studies were assessed to be at unclear risk of bias due to selective reporting as studies were not pre-registered and insufficient information was available. All studies were assessed to be at unclear risk of bias. Figure 4 shows a visual depiction of the risk of bias assessment for each study using three colours to indicate different levels of bias risk: red = high, yellow = unclear, and green = low.

### Publication bias

For the comparison of the intervention and control group on global cognitive functioning measures, the trim-and-fill procedure identified one study with an effect smaller than the mean which modified the results (*g* = 0.89, 95% CI [0.37, 1.41]). However, no major changes in Hedges *g* were observed after the trim-and-fill procedure was conducted as the effect was still large, and the 95% CI remained within similar margins (see Table 5).

**Table 5:** Observed values and the adjusted values for global cognitive functioning after the trim-and-fill procedure

	Studies trimmed	Point estimate	95% CI lower limit	95% CI upper limit	*Q* value
Observed value	–	1.06	0.58	1.54	20.85
Adjusted value	1	0.89	0.37	1.41	31.19

## Discussion

The present review and meta-analysis aimed to assess the effectiveness of immersive VR-based interventions on specific cognitive domains beyond those typically associated with ADHD in children, as well as on global cognitive functioning. The review also aimed to investigate potential moderators of the results and assess the adherence and adverse effects of immersive VR-based interventions versus controls.

To address the first research question that aimed to assess the effectiveness of immersive VR-based interventions in improving specific cognitive domains and general cognition, we performed several analyses. First, we pooled results for attention outcomes, and results showed significant and large improvements for the immersive VR group versus controls for children with ADHD. This is consistent with a previous meta-analysis that found a large effect in favour of immersive VR-based interventions on sustained attention and vigilance measures in children with ADHD compared with controls (Romero-Ayuso et al. 2021). This highlights the potential of immersive VR to treat attention deficits in children with ADHD, one of the primary characteristics of ADHD (APA 2013). Also, one of the included studies included memory outcomes and reported a large effect size in favour of the VR group on memory outcomes, suggesting that immersive VR was significantly more effective in improving memory performance in children with ADHD compared with controls (Tabrizi et al. 2020). This highlights the potential of immersive VR-based interventions to improve cognitive deficits outside of those that primarily characterise ADHD, e.g. memory. Given this was the only study to assess the effectiveness of VR-based interventions on cognitive deficits outside of attention and hyperactivity, this implication should be interpreted with caution.

Results showed large significant improvements on global cognitive functioning between immersive VR and control groups for children with ADHD. VR-based interventions may have the potential to rehabilitate the global cognitive functioning of children with ADHD, and their implementation may have additional benefits. Given the positive association between global cognitive functioning and academic performance (Tikhomirova et al. 2020), and social functioning (Bellanti and Bierman 2000), VR-based interventions may benefit the daily life of children with ADHD in terms of school performance and peer relations. Results are similar to other reviews that investigated if VR can improve attention and short term memory and management of condition among children and youth with ADHD (Rodrigo-Yanguas et al. 2021; Adabla et al. 2021; Peñuelas-Calvo et al. 2022; Goharinejad et al. 2022).

Concerning our second research question that aimed to investigate factors that can improve intervention outcomes, we performed several meta-regressions and subgroup analyses. First, a meta-regression revealed that neither intervention length nor participant age moderated the effect size of global cognitive functioning. The effect of intervention length on the outcomes of VR-based interventions is mixed. Results of previous meta-analyses on children with cerebral palsy (Chen et al. 2014) and people with depression (Legemaat et al. 2021) showed that the length of VR interventions did not moderate the effect size of upper limb activity or depressive symptom severity, respectively. On the other hand, Mekbib et al. (2020) meta-analysis found that VR-based interventions with a length of fifteen hours or greater positively impacted upper limb functioning in stroke patients. It may be possible that both short and long VR-based interventions are sufficiently effective at improving global cognitive functioning, however, given the limited number of studies included, we must interpret this cautiously. This could be in line with the results of previous meta-analyses that showed that treatment duration was a non-significant moderator of behavioural parent training efficacy (Dekkers et al. 2022) as well as of pharmacological treatment efficacy in improving ADHD symptoms (Castells et al. 2021).

Participant age did not moderate the effect size of global cognitive functioning, which is consistent with a previous meta-analysis investigating the effect of cognitive training on children with ADHD who found participant age did not moderate effect sizes of cognitive outcomes (Cortese et al. 2015). In previous meta-analyses that investigated the effectiveness of VR for children without ADHD, participant age has been highlighted as a significant moderator, for example, (Chen et al. 2018) found age to significantly moderate the effect size of physical functioning in children with cerebral palsy after receiving a VR-based intervention. It has been suggested that younger children have more brain plasticity, and therefore have a greater propensity to make larger improvements than adolescents, which may account for the significance of age as a moderator; however, in the current study we did not find support for this.

Other moderators were also tested. Results of the subgroup analysis demonstrated that the type of control did not significantly moderate global cognitive functioning, meaning that there were no differences between type of control groups in the effects they have on the outcomes when compared with immersive VR. Results are in contradiction to previous research that showed larger effects for VR when compared with passive controls versus active controls (Fodor et al. 2018). However, a recent meta-analysis showed similar results with ours, where the VR interventions showed larger effects when compared to active control groups than when compared to passive control groups in improving cognitive functioning in people with mild cognitive impairment (Papaioannou et al. 2022).

Similarly, the diagnostic status of the sample and novelty of VR technology were non-significant moderators. It may be the fact that children without the formal ADHD diagnosis were experiencing similar levels of ADHD symptoms but were not assessed formally by a clinical professional. This could explain why there were no significant differences between the two groups on treatment outcomes. Similar results where formal versus non-formal diagnosis was not a significant moderator of treatment outcomes was reported by Papaioannou et al. (2022) for people with MCI for the comparison between effectiveness of VR versus control interventions. Finally, contrary to previous literature that may have suggested that older HMDs could result in increased simulator sickness and reduced user experience which could have led to reduced performance (Kourtesis et al. 2019) in our study there were no differences between older and newest VR technology on treatment effects.

Moreover, as per our third research question that aimed to assess the treatment adherence and adverse effects of immersive VR versus controls, our results supported the feasibility of immersive VR-based interventions in terms of adherence and safety. Results highlighted that there were no statistically significant differences between groups on participants drop-out rate at the end of treatment. Similar promising results concerning safety of immersive VR emerged, as there were no adverse effects. However, reporting of adverse effects such as simulator sickness is not routinely done, as only two studies out of seven reported any adverse effects for VR and control groups. Both studies, reported no moderate or severe simulator sickness symptoms which is encouraging as there is evidence that simulator sickness could lead to different outcomes as a function of different individual differences (Howard and Van Zandt 2021).

### Strengths, limitations and future directions

This review and meta-analysis has extended previous research by attempting to assess the effectiveness of immersive VR-based interventions for specific cognitive domains beyond the primary cognitive deficits associated with ADHD. Secondly, by compiling all outcome measures for an indication for global cognitive functioning, and finally by conducting moderation analyses. A strength of the review is the rigorous literature search that was conducted according to PRISMA guidelines (Page et al. 2021). Searches were made in major databases with a search strategy devised according to the clinical recommendations from the PICO model. The rigorous literature search means that it is unlikely eligible studies were missed, and thus this review is an accurate synthesis of the present literature. Furthermore, the risk of bias assessment was conducted according to the Cochrane Handbook for Systematic Reviews of Interventions (Higgins et al. 2019), which allowed for a comprehensive analysis of the methodological quality of the included studies and an insight into the impact of study bias on the treatment effects. However, because all studies were at overall unclear risk of bias caused mainly by concerns related to randomisation process and selection of the reported result, results come from potential biased studies and should be interpreted accordingly. We also investigated in a sensitivity analysis if the effect would change in studies where children were withheld treatment during the study. Results showed that in this case the effects would remain significant but larger in magnitude versus the overall effect, suggesting better improvements in cases where children are not taking other treatments.

Finally, the current meta-analysis was the first to address feasibility in terms of treatment adherence and safety of immersive VR in improving cognitive functioning among children with ADHD and showed that VR is both feasible and safe.

There are also limitations of the review to be noted. Firstly, there were few studies included in the meta-analysis, which may reduce the statistical power of the between-groups analysis. This limitation also extends to the moderation analyses, in particular the subgroup analyses where there was a small number of studies in each subgroup.

Overall, the small sample size of the meta-analysis may affect the robustness and reliability of the analysis. The small sample size is unlikely to be the result of a poor literature search, rather as previously observed by Bashiri et al. (2017), research investigating the efficacy of VR interventions for ADHD is scarce. Secondly, the risk of bias assessment highlighted a high risk of bias for all included studies, which also affects the robustness and reliability of the analysis. Due to the small sample size and high risk of bias in the included studies, the findings should be interpreted and reported with caution.

It is recommended that future RCTs assess a broader range of cognitive deficits, which would allow future meta-analyses to assess the effectiveness of VR-based interventions in reducing cognitive deficits of children with ADHD outside of attention and impulsivity (e.g. executive functioning, decision-making and memory). Future RCTs should also attempt to include follow-up measurements so future meta-analyses can assess the long-term effects of VR interventions and whether improvements made on cognitive functioning outcomes from baseline to post-intervention are maintained after the intervention has ceased. This review also highlights the importance of clearly reporting information relating to random sequence allocation, missing outcome data, and analysis plans are given that these domains were judged to be at some risk of bias or high risk of bias for all included studies. Economic outcomes (e.g. cost-effectiveness of immersive VR) were beyond the scope of this review; however, future studies could investigate if immersive VR is cost-effective.

### Implications

Key findings from the current review suggested that immersive VR can be used as an effective tool to improve global cognitive functioning, including attention and memory among children with ADHD. Of extreme importance is the question concerning whether improvements in these cognitive domains can translate to real life. Standardised effects were statistically significant and large in magnitude which suggest the effects are clinically meaningful. Results seem to suggest that in terms of novelty of VR (HMDs) technology, novel HMDs produce similar results as older HMDs; however, this concerns the headset and not the characteristics of the graphics of the VR environment. Improvements in cognition for children in ADHD were observed across all ages and intervention duration, as these variables did not influence the results. Most importantly, the current review brings support in favour of treatment adherence for VR and its safety.

## Conclusions

In summary, this review has demonstrated that immersive VR-based interventions are effective at improving global cognitive functioning, attention, and memory in children with ADHD compared with controls. Moreover, immersive VR is feasible in terms of treatment adherence and a safe cognitive rehabilitation tool. The findings highlight the need for more robust RCTs with clearer reporting of methodology, this will allow for future reviews to draw clear and confident conclusions regarding the effectiveness of VR-based interventions to rehabilitate children with ADHD.

## Data Availability

Data will be made available on reasonable request.
